# From compression to diagnosis: identification of superior vena cava syndrome using point-of-care ultrasound in the emergency department

**DOI:** 10.1186/s12245-024-00597-2

**Published:** 2024-03-13

**Authors:** Noman Ali, Alan Tan, Jordan Chenkin

**Affiliations:** Department of Emergency Medicine, Sunnybrook Health Sciences Centre, 2075, Bayview Ave, Toronto, ON M4N 3M5 Canada

**Keywords:** Point-of-care ultrasound, Mediastinal mass, Superior vena cava syndrome, Lymphoma, Deep venous thrombosis

## Abstract

**Background:**

Superior vena cava (SVC) syndrome is an urgent condition arising from restricted blood flow through the SVC, often linked to factors like malignancy, thrombosis, or infections. Typically, confirmation of the diagnosis involves computed tomography. However, many patients experience respiratory distress and cannot lie supine. Given the increasing integration of point-of-care ultrasound in emergency medicine, it is important to be familiar with findings that are suggestive of this important condition.

**Case report:**

In this case report, we highlight a young patient presenting to the emergency department with superior vena cava syndrome symptoms, successfully diagnosed using point-of-care ultrasound.

**Conclusion:**

This case highlights the utility of point-of-care ultrasound based diagnosis of SVC syndrome and upper arm deep venous thrombosis in a patient with underlying malignancy which ultimately led to early involvement of relevant speciality for initiation of treatment.

**Supplementary Information:**

The online version contains supplementary material available at 10.1186/s12245-024-00597-2.

## Background

Superior vena cava (SVC) syndrome is a collection of signs and symptoms arising from the hindrance of blood circulation in the SVC [1]. Various factors, both malignant and non-malignant, can contribute to SVC syndrome [2]. Notably, lung cancer and non-Hodgkin’s lymphoma account for approximately 90% of cases associated with SVC syndrome [3].

SVC syndrome should be considered a potential diagnosis when a patient exhibits symptoms such as swelling in the upper body, facial redness, distended neck veins, or respiratory difficulty. Imaging is required to identify the underlying cause of SVC obstruction and evaluate the severity of the disease [4]. Over the last decade, point-of-care ultrasound (PoCUS) has increasingly been used to facilitate bedside diagnoses in the emergency department (ED) [5]. PoCUS provides benefits over alternative imaging modalities such as computed tomography (CT), venography, and magnetic resonance imaging, particularly in cases where patients cannot lie flat, receive intravenous contrast, or are too unstable for transfer from the ED to the radiology department.

This case report illustrates a young patient who presented to the ED with a left-sided neck and upper limb swelling. In this case, PoCUS was used to rapidly identify the diagnosis, which resulted in immediate management.

## Case presentation

A 30-year-old woman, previously in good health, visited the emergency department reporting swelling in the left-sided neck veins for one month, along with heaviness and swelling in the left arm for one week. Additionally, she experienced distension of the right-sided neck veins for three days, accompanied by a generalized headache. She had no shortness of breath or chest pain. Notably, she denied experiencing fever, facial swelling, difficulty swallowing, night sweats, or weight loss.

**Fig. 1:**
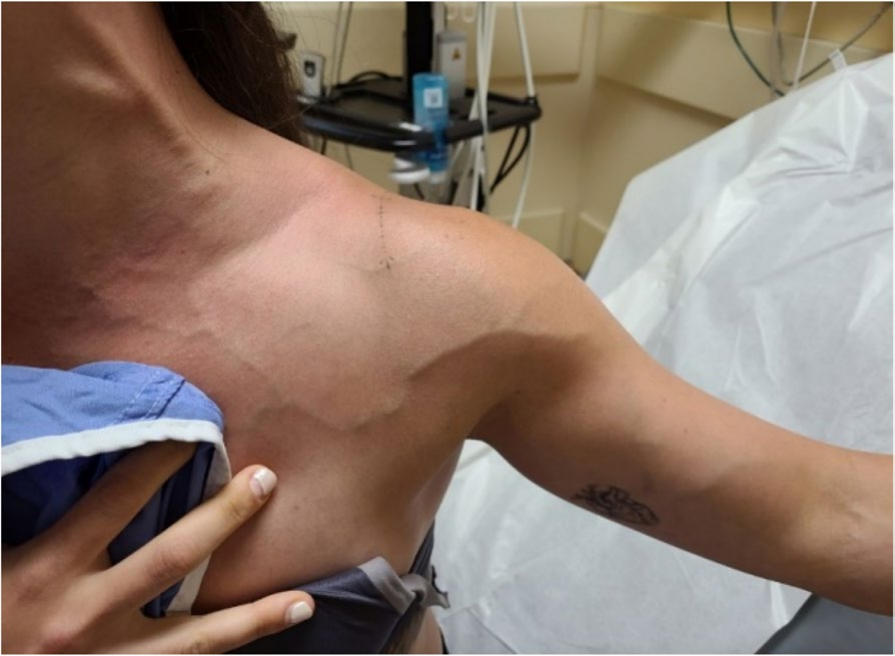
Prominent upper limb veins

On examination, her temperature was 36.8°C, blood pressure was 129/86 mmHg, heart rate was 102 beats per minute, respiratory rate was 18 breaths per minute, and oxygen saturation was 96% on room air. She had visible swelling in the left arm and engorged veins in both arms and neck. Notably, there were no signs of hyperemia, warmth, or tenderness (Fig. 1). The right arm had no visible swelling. The brachial and radial pulses were present and strong in both arms.

**Fig. 2:**
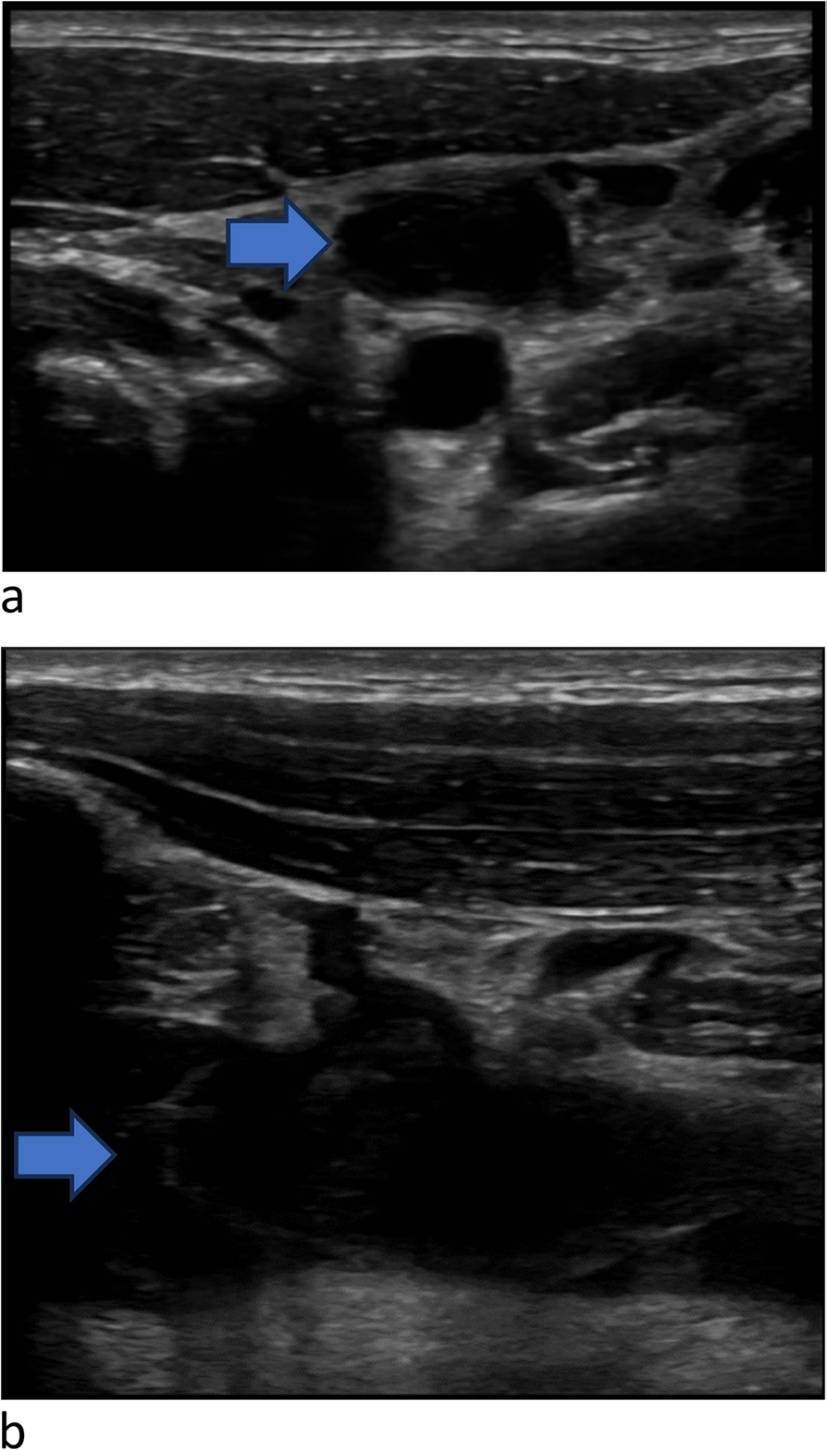
**a** Point-of-care ultrasound showing noncompressible left internal jugular vein (blue arrow) (**b**) Point of care ultrasound showing echogenic clot in the subclavian vein (blue arrow)

**Fig. 3:**
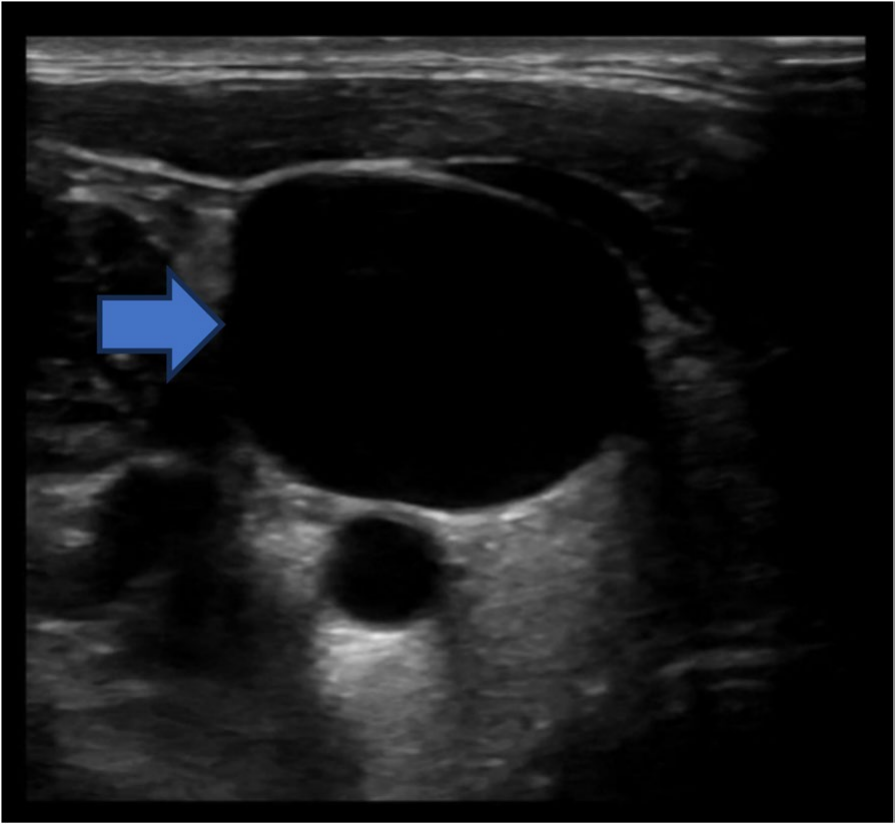
Point of ultrasound showing a very distended right internal jugular vein (blue arrow)

**Fig. 4:**
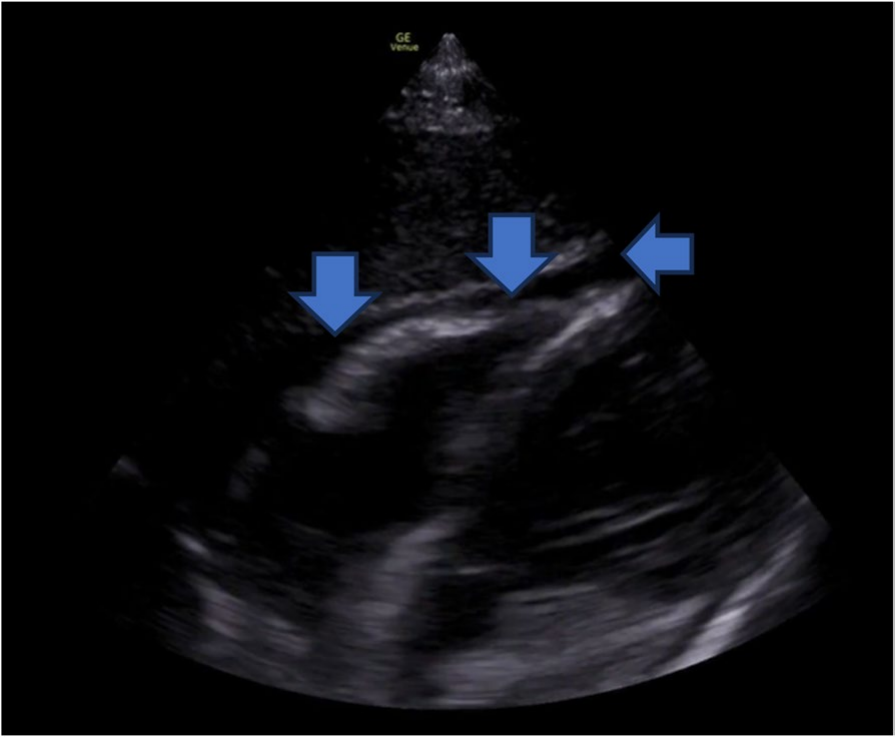
Subxiphoid view of heart showing mild pericardial effusion (blue arrows)

The emergency physician performed a PoCUS of the neck and bilateral upper limb veins. Using a high-frequency linear transducer and with the patient in a semi-recumbent position, the internal jugular (IJ) veins were assessed by gentle compression in B-mode. The subclavian and axillary veins were also evaluated using compression and with colour Doppler. The PoCUS revealed that the left internal jugular vein was noncompressible and displayed echogenic material in the lumen, raising a high suspicion of left internal jugular vein thrombosis (Fig. 2a, Additional file 1, video 1). In addition, thrombus was identified in the left subclavian and axillary veins (Fig. 2b). The right IJ vein did not have any evidence of thrombus, however it was significantly engorged and noncompressible even when the patient was positioned fully upright (Fig. 3, Additional file 2, video 2). In addition to the vascular findings, PoCUS of the heart demonstrated a small pericardial effusion (Fig. 4, Additional file 3, video 3).

**Fig. 5:**
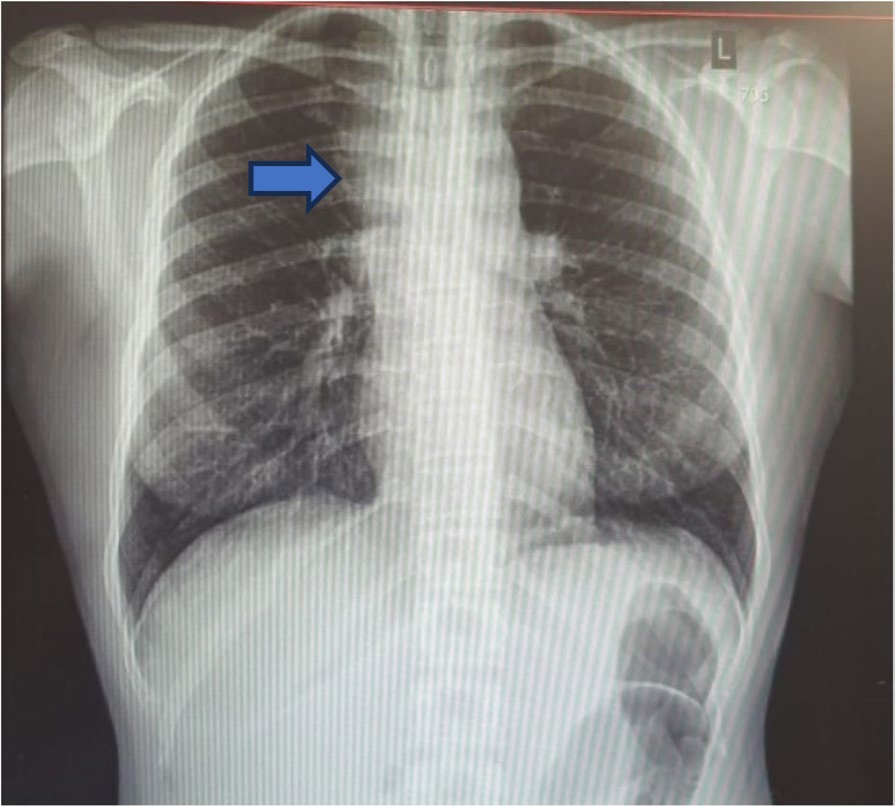
Chest X-Ray showing widened mediastinum (blue arrow)

**Fig. 6:**
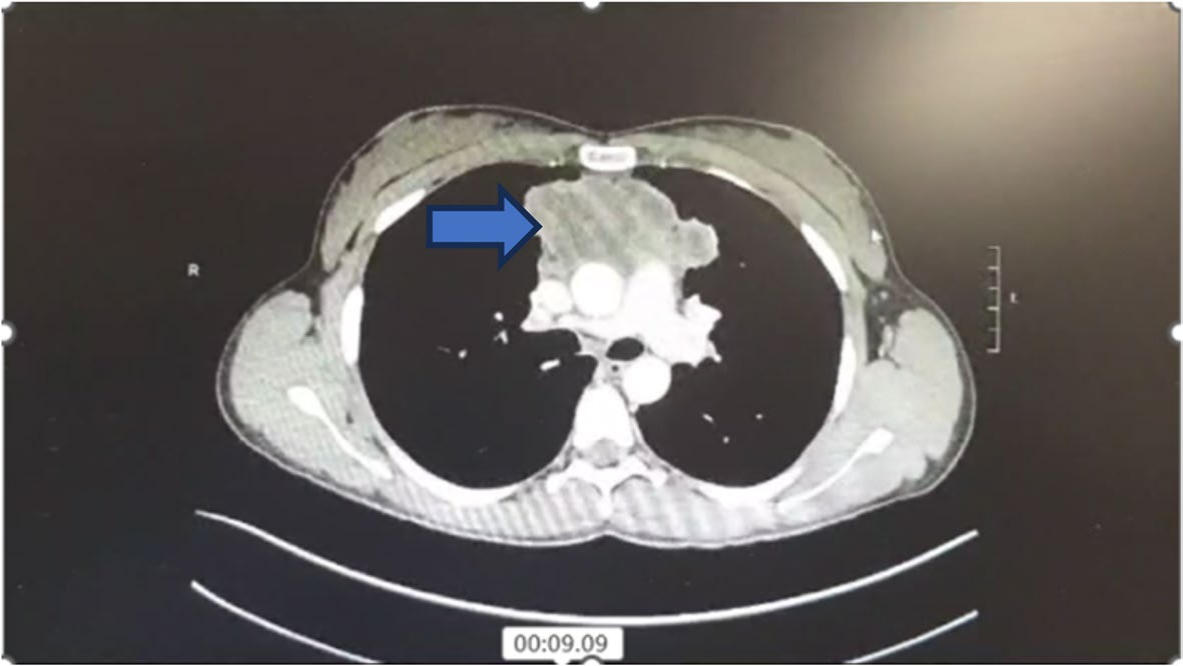
CT-Scan showing mediastinal mass (blue arrow)

A chest X-ray and Doppler ultrasound were ordered. The chest X-ray revealed a widened mediastinum (Fig. 5). A Doppler ultrasound of the neck and bilateral upper extremities veins revealed the presence of an occlusive clot in both the left IJ, subclavian, and axillary veins. Further investigation through a contrast-enhanced CT chest revealed an ill-defined anterior mediastinal mass with diffuse necrosis, extending into the left and right lungs and exhibiting two nodular lesions. This mass measured 6.75 × 5.4 × 8.5cm and exerted pressure on the right brachiocephalic vein and the superior vena cava (Fig. 6).

In response to the thrombotic findings, anticoagulation therapy with enoxaparin was initiated, and the patient was admitted to the internal medicine service for ongoing management. A CT-guided right anterior mediastinal mass biopsy was conducted during the hospitalization. Following successful treatment and stabilization, the patient was discharged with a prescription for direct oral anticoagulants. The biopsy results identified a diffuse large B-cell lymphoma and she was subsequently referred to the oncology clinic where she was started on chemotherapy.

## Discussion

In this case, PoCUS served as an initial step in the diagnostic pathway of SVC syndrome and upper extremity DVT in the ED. The use of PoCUS allowed the correct investigations to be ordered and expedited the diagnosis of an unknown mediastinal mass that required urgent investigation.

SVC syndrome is characterized by swelling of the face, neck, and upper extremities, frequently accompanied by apparent engorged veins in the neck [6, 7]. While upper limb DVT in association with SVC syndrome is uncommon, multiple known risk factors contribute to venous thrombosis. A history of cancer, recent trauma and surgical operations, central venous access, hypercoagulability, and polycythemia are also risk factors [8]. The reduced blood flow through the superior vena cava in SVC syndrome produces an environment conducive to stasis, promoting the formation of clots in the veins of the upper limbs and predisposing individuals to DVT [9].

PoCUS is a useful tool in rapidly identifying the upper limb DVT [10]. The scanning protocol evaluates the IJ, subclavian, axillary, and brachial veins (Additional file 4, Fig. 7 ). A high frequency linear array transducer should be used with the vascular preset. As some of the upper limb deep veins are difficult to compress, the examination may need to rely on finding the echogenic clot and lack of spontaneous or augmented flow using colour Doppler [11]. The transducer should be placed in a transverse position over the sternocleidomastoid muscle to evaluate the IJ vein. The IJ vein should be compressed sequentially down to its confluence with the subclavian vein. Then the transducer should be rotated longitudinally to identify and evaluate subclavian vein below the clavicle. The subclavian vein is difficult to compress and therefore may require colour Doppler evaluation. The subclavian veins should be followed until its bifurcation into the axillary and cephalic veins. Finally, the transducer should be placed in the axilla in transverse to follow the axillary vein and deep brachial veins in the upper arm. These veins should be followed to the antecubital fossa, compressing along their course. In our case, both jugular veins were distended due to compression of the superior vena cava by a mediastinal mass. The stasis of blood within the veins led to the formation of a clot in the left internal jugular and subclavian veins. Distinguishing between distension secondary to SVC compression and clot formation is indeed challenging until echogenic material is observed within the vein. Doppler will be of limited value as there will not be significant velocity of blood flow in either case.

The prognosis of SVC syndrome depends on the underlying cause. CT scan with contrast has traditionally been employed to confirm the diagnosis and provide comprehensive anatomical information about the vena cava and adjacent tissues [12]. Due to the accessibility and non-invasive nature, PoCUS has gained popularity in assessing SVC conditions in recent years. A case report published by Birch et al. demonstrated the utility of PoCUS in rapidly identifying an SVC syndrome associated with bilateral IJ vein thrombosis, facilitating timely clinical interventions in a critically ill patient [13]. Another study conducted by Blanco et al. underscored the utility of PoCUS in detecting mediastinal masses, assessing venous flow abnormalities, and guiding diagnostic interventions, thereby showcasing its potential as a valuable tool in the evaluation of patients presenting with SVC syndrome [5].

## Conclusion

The case emphasizes the utility of PoCUS to assess a patient with suspected SVC syndrome in the emergency department. The finding of significantly distended veins along with consistent symptoms suggests the diagnosis of SVC syndrome and should prompt further investigation. A noncompressible internal jugular vein with echogenic material suggests the presence of thrombus. These findings can help expedite additional investigations and allow early initiation of treatment.

### Supplementary Information

**Additional file 1.** Non-compressible left internal jugular vein with thrombus.

**Additional file 2.** Non-compressible left internal jugular vein with thrombus.

**Additional file 3.** Subxiphoid cardiac view with a small pericardial effusion.

**Additional file 4.** Deep veins anatomy of upper limb.

## Data Availability

No datasets were generated or analysed during the current study.
