# Supplementary figures and images for: From compression to diagnosis: identification of superior vena cava syndrome using point-of-care ultrasound in the emergency department
Source: Int J Emerg Med. 2024 Mar 13;17:40. doi: 10.1186/s12245-024-00597-2 (PMC10935794; doi:10.1186/s12245-024-00597-2)

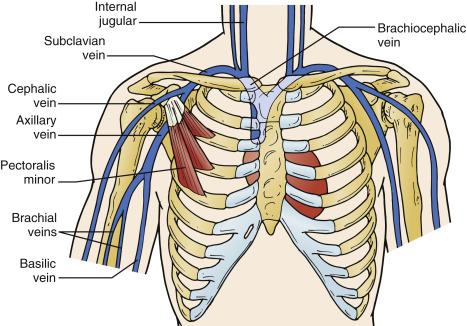

Supplement: Supplementary file 4 — Additional file 4. Deep veins anatomy of upper limb. [file 12245_2024_597_MOESM4_ESM.jpg]
